# Influence of Long-Term Soccer Training on the Fatty Acid Profile of the Platelet Membrane and Intra-Platelet Antioxidant Vitamins

**DOI:** 10.3390/nu16152391

**Published:** 2024-07-23

**Authors:** Víctor Toro-Román, Jesús Siquier-Coll, Ignacio Bartolomé, Marcos Maynar-Mariño, Francisco J. Grijota

**Affiliations:** 1 Research Group in Technology Applied to High Performance and Health, Department of Health Sciences, TecnoCampus, Universitat Pompeu Fabra, 08302 Mataró, Spain; vtoro@tecnocampus.cat; 2 Department of Communication and Education, University of Loyola Andalucía, 41704 Sevilla, Spain; 3 Education Faculty, Pontifical University of Salamanca, Henry Collet Street, 52-70, 37007 Salamanca, Spain; 4 Department of Health Sciences, Universidad Isabel I, Fernán González Street, 76, 09003 Burgos, Spain; fgrijotap@unex.es; 5 Sport Sciences Faculty, University of Extremadura, Avenida de la Universidad s/n, 10003 Cáceres, Spain; mmaynar@unex.es

**Keywords:** oxidative stress, training, lipids, peroxidation, desaturation, vitamin C, vitamin E

## Abstract

This research aimed to study the long-term effects of soccer training on platelet membrane fatty acid levels and antioxidant vitamins. Forty-four subjects divided into soccer players (SP; n = 22; 20.86 ± 0.36 years) and a control group (CG; n = 22; 21.23 ± 0.49 years) participated in the study. The fatty acids of the platelet membrane, the rates of desaturation, lipid peroxidation indexes and intra-platelet levels of vitamins C and E were assessed. SP obtained lower values in polyunsaturated fatty acids 18:3:3 (alpha-linolenic acid), 20:5:3 (eicosapentaenoic acid) and 22:6:3 (docosahexaenoic acid) (*p* < 0.05). The desaturation index ∆5 was higher in SP (*p* < 0.05), and they had a higher lipid peroxidation index 20:4:6 (arachidonic acid)/16:0 (palmitic acid) (*p* < 0.05). Vitamin E and C platelet values were also higher in SP (*p* < 0.01). There were positive correlations in the ω6/ω3 index (*p* < 0.05), desaturation index ∆5 (*p* < 0.05), lipid peroxidation index 20:4:6/16:0 and intra-platelet vitamins E and C (*p* < 0.01) with the level of physical activity. In addition, there were inverse correlations in fatty acids 24:0 (lignoceric acid), 16:1 (palmitoleic acid), 20:3:6 (eicosadienoic acid) and 18:3:3 (alpha-linolenic acid) (*p* < 0.05) depending on the degree of physical activity. Regular long-term soccer training could modify the concentration of fatty acids such as 24:0, 16:1, 18:6, 20:3:6, 18:3:3:3, 20:5:3, 26:6:3 and ω3 PUFAs in the platelet membrane.

## 1. Introduction

Fatty acids are essential in the structure of cell membranes [1], being part of molecules that constitute approximately 50% of these membranes as phospholipids and glycolipids [2]. The lipid bilayer is the basic structure of cell membranes and organelles. The fluid properties of layers are indispensable for many cellular functions. Slight changes could cause an abnormal function in the cell. These characteristics are mainly determined by the presence of polyunsaturated fatty acids (PUFAs) in phospholipid molecules located on both sides of the lipid bilayer [3].

Oxidative stress produced in cells due to an imbalance between prooxidant/antioxidant systems causes alterations in nucleic acids, proteins, carbohydrates and structural lipids [4]. This oxidative state increases the production of reactive oxygen species (ROS) and free radical species [5]. ROS has high reactivity against PUFAs. This process could damage cell membranes and lipoproteins.

Lipid peroxidation (LP) is the main consequence of cell deterioration. This phenomenon is caused by free radicals, affecting the membrane phospholipids [6]. The peroxidation process involves a chain reaction which oxidizes fatty acid, which becomes radical, and is able to oxidize adjacent molecules [7]. Free radicals stimulate the chain reaction of LP by extracting hydrogen atoms [8]. This process could alter the membrane assembly, resulting in changes in fluidity, permeability, alterations in ion transport and the inhibition of metabolic processes [9].

It is known that physical exercise generates the appearance of ROS and free radicals [10]. This phenomenon induces LP [11]. Increased oxygen consumption, the release of catecholamines, the activation of enzymes such as xanthine oxidase or the generation of free radicals by mitochondria are factors that can increase the appearance of ROS [12]. It has been reported that a single physical exercise session induces oxidative stress and elevates the activities of antioxidants [13]. Similarly, the practice of regular physical exercise can attenuate the oxidative stress induced by increasing antioxidant levels [14]. Additionally, this elicits a drop in LP levels [15]. Therefore, the acute effect of physical exercise induces oxidative stress and the appearance of ROS. However, regular physical exercise stimulates the endogenous antioxidant system and protects the body against the adverse effects of oxidative damage [16].

The effect of regular exercise on membrane lipids in erythrocytes seems clear [17,18]. Similarly, the influence of diet on membrane lipids in adipocytes has also been extensively studied [19,20]. Other research has investigated the effects of acute and regular exercise on enzymatic and non-enzymatic antioxidants [21]. Nevertheless, understanding the impact of exercise on membrane lipids is still incomplete.

Platelets are associated with hemostasis and flow very near to the vessel wall to perform this function. This proximity allows for a rapid response when a vascular lesion occurs [22]. The half-life of fatty acids in adipose tissue is approximately 680 days [23], while erythrocytes last 120 days [24] and platelets 9–10 days [25]. Therefore, platelets can provide a more up-to-date evaluation of the effects of physical exercise on body cells.

Previous studies have reported information on membrane fatty acids and their relationship with oxidative stress in healthy subjects [26], after myocardial infarction [27] and with lung cancer [28]. However, there is no information in athletes. Thus, this research aims to study the chronic effects of soccer training on the levels of platelet fatty acids, desaturation rates, lipids peroxidation and non-enzymatic antioxidant systems in soccer player and control subjects and to identify the possible relationships between the properties of the platelet membrane and the degree of physical activity. We hypothesized that regular physical exercise would change the profile of fatty acid in platelets, improving their function.

## 2. Materials and Methods

### 2.1. Participants

Forty-four subjects belonging to the geographical area of Extremadura, divided into two groups: soccer players (SP; 20.86 ± 0.36 years; 1.78 ± 0.54 m; 69.86 ± 4.53 kg) and a control group (CG; 21.23 ± 0.49 years; 1.68 ± 0.39 m; 72.10 ± 8.56 kg), participated in the present experimental study. All of them were informed about the purpose of the study and signed a consent form before enrolling in the study. The protocol was reviewed and approved by the Biomedical Ethics Committee of the University of Extremadura (Spain) and followed the guidelines of the Helsinki Declaration of Ethics for research with human subjects (76/2015). The inclusion criteria were to be a man, aged in the range 18–25 year, residing in the same geographical area, not to be following any particular diet or taking vitamin/mineral or specific supplements, not to have modified the diet for two months before the start of the study and not to suffer any disease during the intervention. The exclusion criteria were the following: (i) taking medications that influence platelet metabolism; (ii) smoking; (iii) having any type of pathology; (iv) having modified diet 10 days before the study.

**Figure 1:**
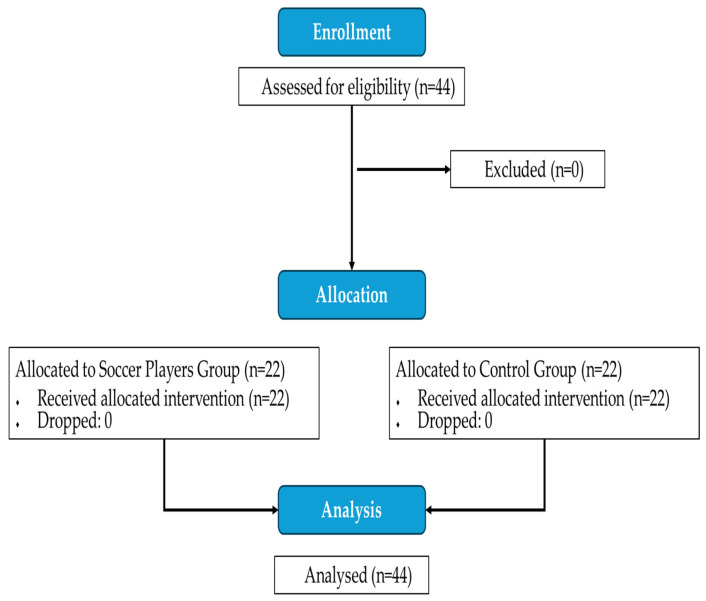
Consort flow diagram.

The SP were players from a third division amateur team. For the previous 2 years, the SP conducted 5 weekly 90 min training sessions. The CG were people who did not perform regular physical exercise. Participants answered a short version of the IPAQ questionnaire translated into Spanish [29] to record the hours of physical activity. In the case of SP, the subjects belonging to this group performed 48.5 ± 5.3 MET-hours/week of physical activity and had experience of at least seven years. On the other hand, in the CG, the subjects performed 22.1 ± 4.1 MET-hours/week of physical activity. The study was carried out after 3 days of inactivity by trained subjects to avoid the influence of the acute effect of the practice of physical exercise. Figure 1 shows the consort flow diagram.

### 2.2. Study Design

**Figure 2:**
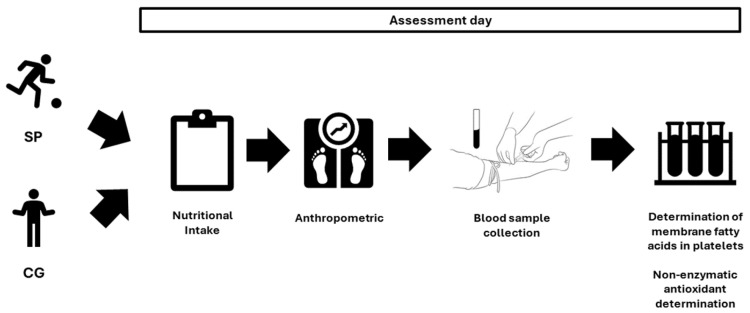
Study design; SP: soccer players; CG: control group.

In the present cross-sectional quasi-experimental study, assessments were performed in the following order: nutritional intake, anthropometry, blood sample collection, and determination of platelet membrane fatty acids and non-enzymatic antioxidants (Figure 2).

### 2.3. Nutritional Intake

The nutritional composition of each food was evaluated. Participants were given a document in which they had to indicate the amount and frequency of food intake during the 3 days prior to the assessment. The investigators conducted a food conversion to estimate consumption using predetermined tables [30].

### 2.4. Anthropometric

The anthropometric characteristics were measured in the morning in fasting conditions by the same investigator after blood samples were drawn, using a Seca © brand scale (Seca 769, Seca, Hamburg, Germany), with an accuracy of ±100 g; a height rod of the Seca © brand (Seca 220, Seca, Hamburg, Germany), with an accuracy of ±1 mm; a Holtain © skin fold caliper (Crymych, UK), with an accuracy of ±0.2 mm; a Holtain © bone diameter caliper (Crymych, UK), with an accuracy of ±1 mm; and a measuring tape of the Seca © brand (Hamburg, Germany) with an accuracy of ±1 mm. The muscle, fat and bone percentages were calculated with equations from the Spanish Kinanthropometry Group [31]. Height, weight, skinfolds (abdominal, suprailiac, subscapular, tricipital, thigh and leg), bone diameters (biestyloid, humeral biepicondyle and femoral biepicondyle) and muscle perimeters (relaxed arm and relaxed leg) were measured.

### 2.5. Determination of Membrane Fatty Acids in Platelets

This section is similar to that presented by Muñoz et al. [32] and Iglesias et al. [33] for the determination of fatty acids.

Blood samples were taken from the antecubital vein in 10 mL glass tubes with ethylenediamine tetraacetate (EDTA) after a nighttime fasting period of at least 10 h. The blood was immediately centrifuged at 1800 rpm for 8 min, then the platelet-rich plasma (PRP) near the erythrocytes was removed and that PRP was centrifuged at 3000 rpm for 10 min. The plasma was removed and 0.5 mL of Mili Q water was added to the platelets.

Free fatty acid concentrations were determined in platelets using the technique described by Lepage and Roy [34]. A gas chromatograph HP-5890 Series II (Hewlett-Packard Company, Marshallton, Delaware, EE. UU.) was used, with a flame ionization detector (FID). The column used was a BPX70 capillary column of 50 m × 0.22 mm I.D., with a film thickness 0.25 μm, Cromlab (Barcelona, Spain). The initial oven temperature was set at 140 °C and held for 15.0 min. It was gradually increased to 190 °C for 15.0 min at a rate of 3 °C/min and to 245 °C at 3 °C/min. The final temperature was maintained for 30.0 min. The inert gas used was helium (He) at a flow rate of 1.0 mL/min. The quantity of FAME analyzed should not exceed 60 ng individually or 150 ng in total to avoid overloading the column. The injector was used in splitless mode, its temperature set at 300 °C and a purge flow of 6 mL/min was applied 0.5 min after the injection. FID was used and set at 250 °C.

The identification of fatty acids was carried out by comparing the retention times of the methyl derivatives of the fatty acids studied, with the fatty acid patterns in the same chromatographic conditions and using retention parameters relative to the internal standard. The internal pattern chosen was heptadecanoic acid, because it is a similar substance to those analyzed and it is well located in the chromatogram, which did not interfere with other peaks in the sample. After obtaining fatty acid values, the ω3 and ω6 peroxidation indexes shown in Table 1 were calculated through PUFA and saturated fatty acid ratios.

After obtaining the percentages of fatty acids, indexes were calculated between the fatty acids shown in Table 1, according to Halliwell and Gutteridge [35].

The ω6/ω3 index expresses the coefficient between the total fatty acids ω6 and the ω3, which is related to the stability of the cell membrane [36].

The desaturation index (∆) refers to the performance of desaturases, enzymes responsible for the desaturation of fatty acids forming double bonds. Therefore, these molecules play an important role in the conversion of saturated fatty acids into unsaturated ones. Elsewhere, the LP indicates the breakdown of long-chain fatty acids in others of short-chain as a result of oxidative damage [35].

### 2.6. Non-Enzymatic Antioxidant Determination

High-pressure liquid chromatography (HPLC) was used for the determination of vitamin C according to the technique described by Manoharan and Schwille [37]. One hundred microliters of 10% perchloric acid mixed with 1% metaphosphoric acid was added to 200 μL of platelets, stirred in a vortex for 30 s and stored in a refrigerator for 20 min. Subsequently, 200 μL of mobile phase was added and centrifuged at 12,000 rpm for 2 min. Twenty microliters of supernatant was collected and injected for vitamin C determination by HPLC. The column used was a C18 11 cm long with a 4. 7 mm internal diameter, using as mobile phase ammonium phosphate 20 mM: 0.015% metaphosphoric at a flow of 1 mL/minute. Detection was performed at a wavelength of 240 nm. The platelet concentration in μg/mL of vitamin C was calculated using the sample dilution factor and a straight line constructed with commercial ascorbic acid.

HPLC was used in the same way as previously described by Lim [38] for the determination of vitamin E. An internal standard of 100 μL of acetate–tocopherol αin ethanol (50 mg/L) was added to 200 μL of platelets and stirred in a vortex for 30 s. Subsequently, 200 μL of n–hexane was added and stirred again for another 30 s, and then mechanically stirred for 10 min to centrifuge at 12,000 rpm for 5 min. Once the centrifugation was finished, the upper layer was removed and dried in a stream of N2 at 37 °C. Immediately before measuring by HPLC, it was reconstituted in 100 μL of ethanol, injecting 20 μL for vitamin E determination. The column used was a Brownlee OD-MP 10 cm long with a 4.6 mm internal diameter, using as mobile phase dichloromethane in 7% methanol (*v*/*v*) at a flow of 1 mL/minute. Detection was performed at a wavelength of 292 nm. The platelet concentration in μg/mL of vitamins A and E was calculated by comparing the areas with the internal pattern.

### 2.7. Statistical Analysis

The statistical analysis was performed with IBM SPSS Statistic 23.0 software for Windows and following Pallant’s guidelines [39]. The results are presented as mean ± standard deviation. Normality was analyzed with the Shapiro–Wilk test, and Leven’s test was used to determine homogeneity of the variances. Mann–Whitney’s U test was used to compare values between groups. Pearson’s correlation coefficient was employed to determine the relation among the variables. A *p* < 0.05 was considered statistically significant.

## 3. Results

Table 2 shows the differences in body composition between SP and CG. Significant differences in abdominal, suprailiac, tricipital (*p* < 0.01) and subscapular (*p* < 0.05) skinfolds were observed between groups. Leg perimeter and muscle percentage were higher in SP with respect to CG (*p* < 0.01; *p* < 0.05). In addition, fat percentage was lower in SP in comparison to CG (*p* < 0.05).

Table 3 shows the nutritional intake of both groups. SP ingested more energy (*p* < 0.05).

Table 4 reflects the percentages of intraplatelet fatty acids divided by groups. There were lower values of PUFAs ω3 18:3:3, 20:5:3 and 22:6:3 (*p* < 0.05) in SP. Similarly, reduced concentrations of 24:0 were observed in SP (*p* < 0.05). Regarding PUFAs ω6, there were higher values of 18:2:6 (*p* < 0.05) in SP, while 20:3:6 was lower than CG (*p* < 0.01). PUFAs ω3 fatty acids were lower in SP (*p* < 0.05).

**Figure 3:**
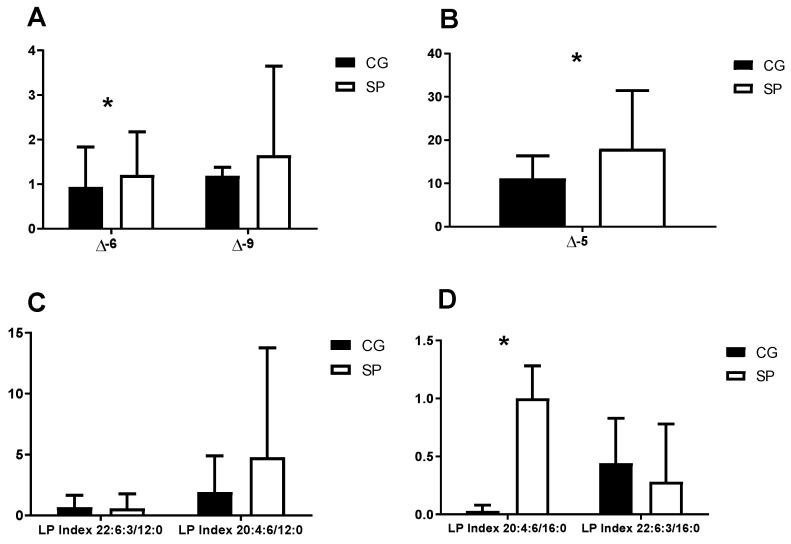
(**A**) values of the desaturation indexes ∆6 and ∆9 in platelets of SP and CG subjects; (**B**) desaturation index values ∆5 in platelets of SP and CG subjects; (**C**) values in areas of the 22:6:3/12:0 and 20:4:6/12:0 indexes of platelet lipid peroxidation in SP and CG subjects; (**D**) values of the 20:4:6/16:0 and 22:6:3/16:0 indexes of platelet lipid peroxidation in SP and CG subjects; *: *p* < 0.05; SP: soccer players; CG: control group; LP: lipid peroxidation; ∆: delta.

Figure 3 presents the values of the delta desaturation indexes and the lipid peroxidation areas of platelet membrane fatty acids in the different groups studied. Significant differences were detected in the ∆5 and ∆6 indexes (Figure 3A,B), being higher in the SP subjects with respect to the CG (*p* < 0.05). Regarding the lipid peroxidation index, a significant difference was observed in the value of the area of the LP 20:4:6/16:0 (Figure 3D), being higher in the SP (*p* < 0.05).

**Figure 4:**
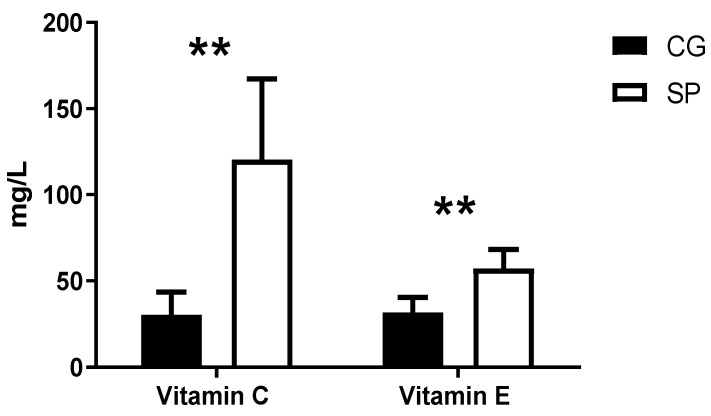
Values of intra-platelet vitamins C and E in soccer players and control subjects. **: *p* < 0.01; SP: soccer players; CG: control group.

Figure 4 shows the values obtained from the intra-platelet antioxidant vitamins C and E. A higher concentration of vitamins E and C was found in SP (*p* < 0.01).

Table 5 presents the data referring to correlations according to physical activity and the different fatty acids studied, as well as the peroxidation rates and antioxidant vitamins studied. A high positive correlation can be observed between the indexes ω 6/ω3 (*p* < 0.05), ∆5 (*p* < 0.05), the index of lipid peroxidation 20:4:6/16:0 (*p* < 0.05) and intraplatelet vitamins E and C (*p* < 0.01) with the physical activity degree. On the other hand, reverse correlations were obtained between fatty acids 24:0, 16:1, 20:3:6 and 18:3:3 and the degree of physical activity (*p* < 0.05).

## 4. Discussion

There are some studies on the fatty acid profile in erythrocytes of athletes [17,18,40,41,42,43]. However, to our knowledge, no studies on the difference in platelet membrane fatty acid and vitamin antioxidant profile have been reported. Our study showed that soccer players differed in their platelet membrane lipid and vitamin antioxidant profiles when compared to a sedentary population.

Table 1 shows the anthropometric differences between groups, with a lower fat percentage and a higher muscle percentage in SP. Similarly, a recent study analyzed the chronic effect of different types of training for 12 months in adults with different body mass indexes, resulting in increases in muscle mass and decreases in fatty tissue [44]. Other research investigated the chronic effect of Crossfit practice, with 70 min sessions, three days per week for 14 weeks in university students, observing changes in muscle mass and body fat [45]. This low percentage of fat and the higher muscle percentage in the SP in this study could be related to the an increase in lipid metabolism as a source of energy [46,47].

The results obtained in intraplatelet fatty acids are discrepant. Despite the absence of differences in the nutritional intake of fatty acids and vitamins, there was a lower concentration of ω3 fatty acids in SP. This was accompanied by a higher concentration of ω6 fatty acids. Similar to previous studies, in the present investigation, C16:0 was one of the major fatty acids in the platelet membrane [26].

It is known that regular physical training induces changes in the composition of fatty acids. Thus, Helge et al. [48] observed changes in the phospholipids of the muscle membranes after four weeks of training in one leg, increasing the content of 18:1 and 22:6:3. Also, Tepsic et al. [41] analyzed the fatty acid profile of erythrocyte phospholipids in amateur boxers, observing a significant decrease in SFAs and MUFAs compared to the control group. However, in PUFAs ω3 and ω6, there were only significant differences in 18:2:6, which was lower in the group of boxers. Other research analyzed the fatty acids of phospholipids in erythrocytes in basketball and soccer players and controls. The profile of erythrocyte fatty acids showed no differences between soccer players and controls. However, basketball players had a higher ratio of 18:0 than controls, lower 18:2 than the other two groups and higher 22:4 than soccer players [18]. Therefore, long periods of exercise and the type of sport can influence fatty acid profiles.

It is known that the fatty acids in the various membranes of the body come from the same pool of fatty acids (plasma) which in turn comes mainly from dietary fats [49]. The uptake of fatty acids from plasma is specific to the different tissue types and depends on their function. It has been shown that there are different families of fatty acid binding proteins in humans, with different fatty acid binding capacities [49]. Therefore, differences could exist when comparing erythrocyte and platelet membrane fatty acids. Diet can also influence the composition of fatty acids [50]. The effects of fatty acid intake on the platelet membrane have been extensively studied [51,52]. Therefore, for this investigation, the participants had not to have altered their diet in previous years and had to live in the same geographical area to be eating a similar diet.

The scientific literature on the influence of regular physical exercise on aggregation and platelet function is scarce. The studies have been focused on the effect on number and volume in healthy individuals [53,54,55]. These studies have shown that moderate-intensity physical exercise in young men and women reduces the adhesion, aggregation and number of platelets at rest and after physical exercise. Physical exercise seems to stimulate the production of prostacyclin, a potent inhibitor of platelet aggregation, and the production of nitric oxide, a potent antiplatelet [56]. Additionally, the unfavorable adverse effects associated with platelet aging appear to be attenuated with physical exercise [57]. Regular exercise can reduce the risk of vascular thrombotic events and protect against cardiovascular disease. These changes are likely to be mediated by the improvement in the bioavailability of nitric oxide in platelets and the potential for the release of nitric oxide derived from platelets after physical training [58].

We hypothesized that regular physical exercise would change the profile of fatty acid in platelets, improving their function. The production of eicosanoids in platelets from PUFAs ω3 in SP could improve their physiological functions. The content of PUFAs in platelets depends on the diet. However, this content also depends on the ability of enzyme desaturases and elongases to act on linoleic and linolenic acid and transform them into arachidonic acids (AAs) and eicosapentaenoic acids (EPAs). These fatty acids can be deposited in cell membranes and used in endothelial cells to produce eicosanoids [59].

EPA and AA linked to the action of phospholipase A_2_ produce two types of eicosanoids: thromboxanes (TXs) and prostaglandins (PGs) [60,61]. Platelets produce a high number of TX due to their high content in the enzyme thromboxane synthetases. The TXs derived from AA have vascular vasoconstrictor and proaggregating actions [62]. EPA-derived TXs are inactive. The PGs are divided into PG_2_ and PG_3_. PG_2_ is produced from AA and is antiplatelet and vasodilator [63]. PG_3_ derived from EPA has similar properties to PG_2_ [64]. The intake of AA generates vasoconstrictor, proaggregant and proinflammatory [26,65] eicosanoids in platelets. EPA-derived eicosanoids are vasodilators and anti-inflammatory [66]. This would be accompanied by an increase in platelet membranes of the ω6/ω3 ratio and a rise in the desaturation indexes ∆5 and ∆6 in GE. The lower concentrations of ω3 fatty acids observed in SP could be due to a predilection of platelet phospholipase A2 for ω3 derivatives induced by physical training. The higher use and production of ω3 fatty acids would lead to less aggregative and low vasoconstrictor power in platelets. This would elicit an improvement in the functions of membrane phospholipid remodeling reactions [67].

We understand that the changes produced in the platelet membranes are due to an adaptive process, due to training, which would lead to an anti-inflammatory state in the athlete and an improvement in the fluidity of these membranes that would improve the response to the signals marked by the exerkines.

There is higher peroxidation in the 20:4:6/12:0 and 20:4:6/16:0 indexes in the SP compared to the CG. However, the lipid peroxidation rate 22:6:3/16:0 was higher in the CG. Lipid peroxidation was much more intense in healthy individuals. These data could be explained by a higher amount of PUFAs in the platelet phospholipid membrane [26,65]. Previous authors observed lower values of fatty acid lipid peroxidation in the indexes 22:6:3/12:0 and 20:4:6/12:0 in the erythrocyte membrane in trained subjects compared to sedentary subjects [33]. It seems that physical exercise is associated with an increase in the mobilization of PUFAs of the membranes. This phenomenon would result in a subsequent insertion of SFAs for the regeneration of membrane lipids due to the lipid peroxidation processes produced by physical exercise [32].

Likewise, the evaluation of antioxidant vitamins C and E indicates the presence of higher concentrations of these antioxidants in the SP compared to the CG. Similarly, Brites et al. [68] observed high levels of vitamin E and C in plasma in football players who trained regularly compared to the control group. Conversely, Siquier et al. [69] analyzed the chronic effect of physical exercise in soccer players, observing lower values in intra-red cell antioxidant vitamins in football players than in controls. Elsewhere, Muñoz et al. [70] obtained significantly higher levels of vitamin C in plasma and significantly lower ones in erythrocytes after a maximum test in trained cyclists. This controversy in the results may be due to the average life days of each parameter studied. As mentioned before, the half-life of platelets (7–10 days) is shorter compared to erythrocytes (120 days). This leads to longer exposure of free radicals in erythrocytes. This increased exposure would cause a marked decrease in erythrocyte antioxidant vitamins. This deficit of antioxidant vitamins could be due to an increase in the function of these vitamins to combat oxidative stress derived from chronic training to maintain a homeostatic state. Therefore, having a shorter half-life, platelets are exposed to free radicals for less time, which can give us more up-to-date information on the athlete’s cellular status. In this study, it can be interpreted that the SP obtained a higher capacity to fight free radicals compared to the CG, due to the antioxidant adaptations produced by the organism as a consequence of regular physical exercise [71]. In our study, the highest concentrations of antioxidant vitamins in athletes would be due to possible adaptations induced by training aimed at improving the absorption and distribution of these vitamins in the athlete since they are subject to a significant oxidative imbalance state and would prevent radicals from free substances from damaging platelets, helping to maintain their proper functioning in this pro-oxidant environment.

The present study is one of the first investigations to analyze the chronic effect of physical exercise on the fatty acid profile of the platelet membrane. Therefore, further research is needed to corroborate the results obtained. The number of participants in the study and the absence of a specific control of the dietary intake of the participants are some of the limitations of the present investigation.

## 5. Conclusions

Regular long-term soccer training could change the profiles of fatty acids in the platelet membrane of soccer players. Changes in membrane fatty acid profiles would be specific adaptations to counteract the negative effect of ROS on athletes.

Intra-platelet levels of vitamin C and E were higher in subjects with a greater degree of training, which represents an increased capacity to defend against ROS. The changes produced could be linked to the optimization of platelet properties and functions that make physical activity have a potent antiatherogenic effect.

The results indicate that the evaluation of platelets could give a more up-to-date view of the chronic effects of physical exercise on body cells and the consequences on their functions due to their short half-life.

## Figures and Tables

**Table 1:** Calculation methods of SFA, MUFAs, PUFAs, desaturation indexes and lipid peroxidation indexes.

SFAs	12:0 (lauric acid) + 14:0 (myristic acid) + 16:0 (palmitic acid) + 18:0 (stearic acid) + 24:0 (lignoceric acid)
MUFAs	16:1 (palmitoleic acid) + 18:1C (oleic acid) + 18:1T (elaidic acid) + 24:1 (nervonic acid)
PUFAs ω6	18:2:6 (linoleic acid) + 18:3:6 (gamma-linolenic acid) + 20:3:6 (eicosadienoic acid) + 20:4:6 (arachidonic acid)
PUFAs ω3	18:3:3 (alpha-linolenic acid) + 20:5:3 (eicosapentaenoic acid) + 22:5:3 (docosapentaenoic acid) + 22:6:3 (docosahexaenoic acid)
PUFAs	PUFAs ω6 + PUFAs ω3
ω6/ω3 index	PUFAs ω6/PUFAs ω3
Desaturation index ∆9	18:1/18:0
Desaturation index ∆6	18:2/18:1
Desaturation index ∆5	20:4:6/20:3:6
Lipid peroxidation index 1	22:6:3/12:0
Lipid peroxidation index 2	20:4:6/12:0
Lipid peroxidation index 3	22:6:3/16:0
Lipid peroxidation index 4	20:4:6/16:0

SFAs: saturated fatty acids; MUFAs: monounsaturated fatty acids; PUFAs: polyunsaturated fatty acids; ω6: omega-6; ω3: omega-3; ∆: delta.

**Table 2:** Results obtained for the 6 skinfolds analyzed (mm), muscle perimeters (cm) and body composition (%).

	SP	CG
Abdominal skinfold (mm)	12.03 ± 3.03	19.01 ± 8.48 **
Suprailiac skinfold (mm)	8.45 ± 2.07	15.77 ± 6.73 **
Subscapular skinfold (mm)	10.09 ± 1.98	14.26 ± 5.87 *
Tricipital skinfold (mm)	9.01 ± 2.82	23.84 ± 38.79 **
Thigh skinfold (mm)	12.55 ± 3.92	15.90 ± 5.51
Gastrocnemius skinfold (mm)	8.43 ± 3.01	9.86 ± 4.06
Arm Perimeter (cm)	27.05 ± 2.00	29.68 ± 2.10
Leg perimeter (cm)	36.87 ± 1.75	35.97 ± 4.65
Fat percentage (%)	9.43 ± 1.08	14.40 ± 6.23
Muscle Percentage (%)	48.05 ± 0.84	44.95 ± 0.85
Bone Percentage (%)	18.42 ± 1.43	17.55 ± 0.57

SP: soccer players; CG: control group; * *p* < 0.05; ** *p* < 0.01 differences SP vs. CG.

**Table 3:** Nutritional intake.

Parameters	SP	CG
Energy (kcal/d)	1796.01 ± 420.37	1504.67 ± 573.21 *
Carbohydrates (g/d)	231.06 ± 69.1	197.24 ± 72.14
Proteins (g/d)	106.13 ± 25.55	87.56 ± 33.12
Fat (g/d)	54.81 ± 19.14	56.56 ± 33.46
Vitamin C (ug/d)	104.95 ± 64.97	102.66 ± 57.14
Vitamin E (ug/d)	8.74 ± 8.72	8.91 ± 7.33
14:0 (myristic acid) (g/d)	1.78 ± 1.13	1.85 ± 1.28
16:0 (palmitic acid) (g/d)	9.75 ± 4.28	10.77 ± 5.10
18:0 (stearic acid) (g/d)	3.74 ± 2.29	4.01 ± 3.21
16:1 (palmitoleic acid) (g/d)	1.28 ± 0.56	1.33 ± 0.87
18:1:C (oleic acid) (g/d)	16.12 ± 6.22	16.56 ± 6.35
18:3:6 (gamma-linolenic acid) (g/d)	6.95 ± 4.50	7.14 ± 5.01
18:3:3 (alpha-linolenic acid) (g/d)	0.90 ± 0.88	0.87 ± 0.92
20:4:6 (arachidonic acid) (g/d)	0.08 ± 0.05	0.09 ± 0.06
20:5:3 (eicosapentaenoic acid) (g/d)	0.25 ± 0.30	0.23 ± 0.27
22:5:3 (docosapentaenoic acid) (g/d)	0.08 ± 0.11	0.08 ± 0.12
22:6:3 (docosahexaenoic acid) (g/d)	0.53 ± 0.65	0.50 ± 0.59

* *p* < 0.05 differences SP vs. CG.

**Table 4:** Differences in platelet membrane fatty acids (%) between SP and CG.

Parameters (%)	SP	CG	*p*
12:0 (lauric acid)	4.43 ± 8.04	1.32 ± 2.61	0.09
14:0 (myristic acid)	0.82 ± 1.04	0.42 ± 0.29	0.09
16:0 (palmitic acid)	14.19 ± 3.26	12.46 ± 3.83	0.11
18:0 (stearic acid)	11.17 ± 3.35	11.35 ± 3.32	0.86
24:0 (lignoceric acid)	1.81 ± 1.41	4.63 ± 3.97	<0.001
16:1 (palmitoleic acid)	0.40 ± 0.28	0.27 ± 0.10	0.04
18:1:C (oleic acid)	13.38 ± 4.05	13.41 ± 3.93	0.98
18:1:T (elaidic acid)	1.06 ± 2.48	0.28 ± 0.38	0.15
24:1 (nervonic acid)	4.97 ± 10.02	5.58 ± 3.07	0.79
18:6 (linoleic acid)	16.21 ± 6.06	12.71 ± 4.06	0.03
18:3:6 (gamma-linolenic acid)	0.73 ± 1.79	0.30 ± 0.20	0.27
20:3:6 (eicosadienoic acid)	1.32 ± 0.61	1.96 ± 0.72	<0.001
20:4:6 (arachidonic acid)	17.83 ± 5.11	18.46 ± 3.07	0.62
18:3:3 (alpha-linolenic acid)	0.76 ± 0.44	1.35 ± 0.50	<0.001
20:5:3 (eicosapentaenoic acid)	0.96 ± 2.62	2.40 ± 2.67	0.04
22:5:3 (docosapentaenoic acid)	4.06 ± 9.82	4.84 ± 3.84	0.73
22:6:3 (docosahexaenoic acid)	2.94 ± 4.15	4.11 ± 2.35	0.04
SFAs	32.43 ± 9.16	30.19 ± 4.07	0.30
MUFAs	19.81 ± 9.44	19.55 ± 2.81	0.90
PUFAs	44.82 ± 9.33	46.15 ± 3.76	0.54
PUFAs ω6	36.10 ± 10.28	33.44 ± 6.13	0.22
PUFAs ω3	8.72 ± 12.65	12.71 ± 8.28	0.04
PUFA ω6/ω3	9.97 ± 9.00	4.56 ± 3.50	0.01

SP: soccer players; CG: control group; SFAs: saturated fatty acids; MUFAs: monounsaturated fatty acids; PUFAs: polyunsaturated fatty acids; ω6: omega 6; ω3: omega 3.

**Table 5:** Correlations between physical activity (MET-hours/week) and SUFAs, MUFAs and PUFAs, ω3/ω6 indexes, desaturation indexes, lipid peroxidation indexes and vitamins E and C in platelets.

	Pearson Correlation (*r*)	*p*
12:0 (lauric acid)	0.26	0.09
14:0 (myristic acid)	0.26	0.09
16:0 (palmitic acid)	0.24	0.11
18:0 (stearic acid)	0.03	0.86
24:0 (lignoceric acid)	−0.44	<0.001
16:1 (palmitoleic acid)	−0.31	0.04
18:1:C (oleic acid)	0.01	0.98
18:1:T (elaidic acid)	0.22	0.15
24:1 (nervonic acid)	−0.04	0.79
18:6 (linoleic acid)	0.25	0.12
18:3:6 (gamma-linolenic acid)	0.17	0.27
20:3:6 (eicosadienoic acid)	−0.44	<0.001
20:4:6 (arachidonic acid)	0.08	0.62
18:3:3 (alpha-linolenic acid)	−0.54	<0.001
20:5:3 (eicosapentaenoic acid)	−0.27	0.08
22:5:3 (docosapentaenoic acid)	−0.05	0.73
22:6:3 (docosahexaenoic acid)	−0.18	0.25
SFAs MUFAs	0.16 0.02	0.30 0.90
PUFAs PUFAs ω6 PUFAs ω3	−0.10 0.16 −0.19	0.54 0.30 0.22
ω6/ω3 index	0.38	0.02
Desaturation index ∆5	0.33	0.03
Desaturation index ∆6	0.24	0.06
Desaturation index ∆9	0.15	0.32
LP Index 22:6:3/12:0	−0.04	0.82
LP Index 20:4:6/12:0 LP Index 22:6:3/16:0 LP Index 20:4:6/16:0 Vitamin C Vitamin E	0.21 −0.14 0.35 0.80 0.79	0.18 0.35 0.02 <0.001 <0.001

SFAs: saturated fatty acids; MUFAs: monounsaturated fatty acids; PUFAs: polyunsaturated fatty acids; LP: lipid peroxidation; ∆: delta.

## Data Availability

Data are contained within the article.
